# 1-{[(2,3-Dihydro-1*H*-inden-2-yl)­oxy]meth­yl}quinazoline-2,4(1*H*,3*H*)-dione

**DOI:** 10.1107/S1600536812022350

**Published:** 2012-05-23

**Authors:** Nasser R. El-Brollosy, Necmi Dege, Güneş Demirtaş, Mohamed I. Attia, Ali A. El-Emam, Orhan Büyükgüngör

**Affiliations:** a Department of Pharmaceutical Chemistry, College of Pharmacy, King Saud University, 11451 Riyadh, Saudi Arabia; b Department of Physics, Faculty of Arts and Sciences, Ondokuz Mayıs University, 55139 Samsun, Turkey

## Abstract

In the title mol­ecule, C_18_H_16_N_2_O_3_, the five-membered ring has an envelope conformation, with the substituted C atom deviating by 0.342 (4) Å from the mean plane *P* calculated for the remainder of the non-H atoms of the 2,3-dihydro-1*H*-indene fragment. The mean planes of quinazoline-2,4(1*H*,3*H*)-dione fragment and *P* form a dihedral angle of 59.08 (4)°. In the crystal, pairs of N—H⋯O hydrogen bonds link mol­ecules into inversion dimers, and weak C—H⋯O hydrogen bonds and π–π inter­actions between the benzene rings of the quinazoline ring systems [centroid–centroid distance = 3.538 (3) Å] further consolidate the packing.

## Related literature

For the biological activity of quinazoline-2,4(1*H*,3*H*)-diones, see: Tran *et al.* (2004 ▶); Cao *et al.* (2010 ▶) and for the biological activity of non-nucleoside reverse transcriptase inhibitors (NNRTIs), see: Hopkins *et al.* (1996 ▶, 1999 ▶); El-Brollosy (2006 ▶, 2007 ▶); El-Brollosy *et al.* (2008 ▶, 2009 ▶). For related structures, see: Liu (2008 ▶); Karimova *et al.* (2010 ▶).
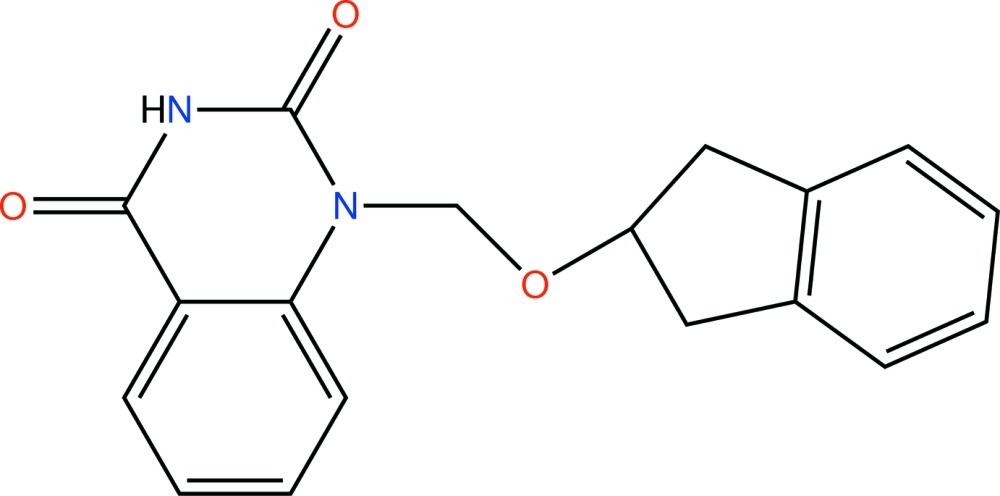


## Experimental

### 

#### Crystal data

C_18_H_16_N_2_O_3_
*M* *_r_* = 308.33
Triclinic, 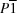

*a* = 7.6684 (8) Å
*b* = 10.0717 (10) Å
*c* = 10.6748 (11) Å
α = 87.199 (8)°
β = 78.332 (8)°
γ = 70.569 (8)°
*V* = 761.28 (13) Å^3^
*Z* = 2
Mo *K*α radiation
μ = 0.09 mm^−1^
*T* = 296 K
0.58 × 0.38 × 0.05 mm

#### Data collection

Stoe IPDS 2 diffractometer
Absorption correction: integration (*X-RED32*; Stoe & Cie, 2002 ▶) *T*_min_ = 0.948, *T*_max_ = 0.995
11601 measured reflections
3156 independent reflections
2078 reflections with *I* > 2σ(*I*)
*R*_int_ = 0.043

#### Refinement

*R*[*F* ^2^ > 2σ(*F* ^2^)] = 0.044
*wR*(*F* ^2^) = 0.103
*S* = 1.00
3156 reflections
208 parameters
H-atom parameters constrained
Δρ_max_ = 0.15 e Å^−3^
Δρ_min_ = −0.16 e Å^−3^

### 

Data collection: *X-AREA* (Stoe & Cie, 2002 ▶); cell refinement: *X-AREA*; data reduction: *X-RED32* (Stoe & Cie, 2002 ▶); program(s) used to solve structure: *WinGX* (Farrugia, 1997 ▶) and *SHELXS97* (Sheldrick, 2008 ▶); program(s) used to refine structure: *SHELXL97* (Sheldrick, 2008 ▶); molecular graphics: *ORTEP-3 for Windows* (Farrugia, 1997 ▶); software used to prepare material for publication: *WinGX* (Farrugia, 1999 ▶) and *PLATON* (Spek, 2009 ▶).

## Supplementary Material

Crystal structure: contains datablock(s) I, global. DOI: 10.1107/S1600536812022350/cv5291sup1.cif

Structure factors: contains datablock(s) I. DOI: 10.1107/S1600536812022350/cv5291Isup2.hkl

Supplementary material file. DOI: 10.1107/S1600536812022350/cv5291Isup3.cml

Additional supplementary materials:  crystallographic information; 3D view; checkCIF report

## Figures and Tables

**Table 1:** Hydrogen-bond geometry (Å, °)

*D*—H⋯*A*	*D*—H	H⋯*A*	*D*⋯*A*	*D*—H⋯*A*
N2—H2⋯O2^i^	0.86	2.06	2.9106 (18)	169
C9—H9*A*⋯O2^ii^	0.97	2.56	3.527 (3)	173
C16—H16⋯O1^iii^	0.93	2.47	3.378 (2)	166
C10—H10*A*⋯O3^iv^	0.97	2.46	3.404 (2)	165
C5—H5⋯O3^v^	0.93	2.47	3.314 (2)	151

Symmetry codes: (i) 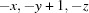; (ii) 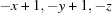; (iii) 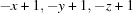; (iv) 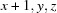; (v) 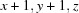.

**Fig. 1.**
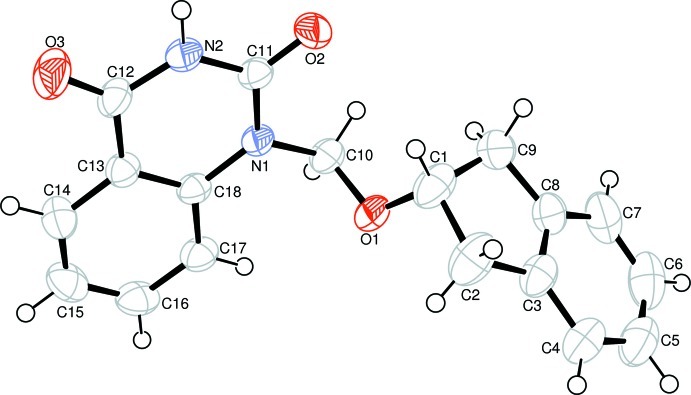
The molecular structure of (I) showing the atomic numbering scheme. Displacement ellipsoids are drawn at the 50% probability level.

## supplementary crystallographic information

### Comment

Non-nucleoside reverse transcriptase inhibitors (NNRTIs) are very promising therapies in the treatment of human immunodeficiency virus (HIV) (Hopkins *et al.*, 1996, 1999). Some series of 3-hydroxyquinazoline-2,4-dione and *N*-((2-methyl-4(3*H*)-quinazolinon-6-yl)methyl)dithiocarbamates have been synthesized and evulated for antibacterial activity (Tran *et al.* 2004; Cao *et al.* 2010). In continuation to our interest in NNRTIs (El-Brollosy *et al.* 2006, 2007, 2008, 2009), we synthesized the title compound, (I), as a potential non-nucleoside reverse transcriptase inhibitor.

In (I) (Fig. 1), in the 2,3-dihydro-1*H*-indene fragment atom C1deviates from the main plane *P* at 0.342 (4) Å. In the literature, some quinazoline-2,4(1*H*,3*H*)-dione structures have been reported (Liu, 2008; Karimova *et al.* 2010). The C11═O2 and C12═O3 bond lengths are 1.2247 (19) and 1.2144 (18) Å, respectively. The C≐C bond distances range from 1.362 (3) Å to1.394 (2) Å. The torsion angle C1—O1—C10—N1 is -93.88 (17)°.

In the crystal, intermolecular N2—H2···O2 hydrogen bond (Table 1) link two molecules into centrosymmetric dimer. Further, weak C—H···O hydrogen bonds (Table 1) and π–π interactions between the benzene rings of the quinazoline bicycles [centroid-centroid distance = 3.538 (3) Å] consolidate the crystal packing.

### Experimental

Quinazoline-2,4(1*H*,3*H*)-dione (162 mg, 1 mmol) was stirred in dry acetonitrile (15 ml) under nitrogen and *N,O*-bis(trimethylsilyl)acetamide (BSA) (0.87 ml, 3.5 mmol) was added. After a clear solution was obtained (10 min), the mixture was cooled down to -50 °C and *TMS* trifluoromethanesulfonate (0.18 ml, 1 mmol) was added followed by the dropwise addition of bis(indan-2-yloxy)methane (560 g, 2 mmol). The reaction mixture was stirred at room temperature for 5 h, and quenched by addition of saturated aqueous sodium hydrogen carbonate solution (5 ml). The mixture was evaporated under reduced pressure and the residue was extracted with ether (3 × 50 ml). The combined ether fractions were dried (MgSO_4_) and evaporated under reduced pressure. The product was purified on silica gel column chromatography, using 20% ether in petroleum ether (40–60°C), to afford the title compound as a white solid in 71% yield (218 mg). Single crystals were achieved by crystallization from ethanol. *M*.p. 193–194 *^o^*C (El-Brollosy, 2007).

### Refinement

All H atoms were positioned geometrically [N—H=0.86 Å; C—H=0.93 Å - 0.98 Å] and treated as riding, with *U*_iso_(H)=1.2*U*_eq_(C, N).

### Crystal data

**Table:**

C_18_H_16_N_2_O_3_	*Z* = 2
*M**_r_* = 308.33	*F*(000) = 324
Triclinic, *P*1	*D*_x_ = 1.345 Mg m^−^^3^
Hall symbol: -P 1	Mo *K*α radiation, λ = 0.71073 Å
*a* = 7.6684 (8) Å	Cell parameters from 11963 reflections
*b* = 10.0717 (10) Å	θ = 2.9–27.9°
*c* = 10.6748 (11) Å	µ = 0.09 mm^−^^1^
α = 87.199 (8)°	*T* = 296 K
β = 78.332 (8)°	Plate, colorless
γ = 70.569 (8)°	0.58 × 0.38 × 0.05 mm
*V* = 761.28 (13) Å^3^	

### Data collection

**Table:**

Stoe IPDS 2 diffractometer	3156 independent reflections
Radiation source: fine-focus sealed tube	2078 reflections with *I* > 2σ(*I*)
Graphite monochromator	*R*_int_ = 0.043
rotation method scans	θ_max_ = 26.5°, θ_min_ = 2.9°
Absorption correction: integration (*X-RED32*; Stoe & Cie, 2002)	*h* = −9→9
*T*_min_ = 0.948, *T*_max_ = 0.995	*k* = −12→12
11601 measured reflections	*l* = −13→13

### Refinement

**Table:**

Refinement on *F*^2^	Primary atom site location: structure-invariant direct methods
Least-squares matrix: full	Secondary atom site location: difference Fourier map
*R*[*F*^2^ > 2σ(*F*^2^)] = 0.044	Hydrogen site location: inferred from neighbouring sites
*wR*(*F*^2^) = 0.103	H-atom parameters constrained
*S* = 1.00	*w* = 1/[σ^2^(*F*_o_^2^) + (0.0506*P*)^2^] where *P* = (*F*_o_^2^ + 2*F*_c_^2^)/3
3156 reflections	(Δ/σ)_max_ < 0.001
208 parameters	Δρ_max_ = 0.15 e Å^−^^3^
0 restraints	Δρ_min_ = −0.16 e Å^−^^3^

### Special details

**Table:**

Geometry. All e.s.d.'s (except the e.s.d. in the dihedral angle between two l.s. planes) are estimated using the full covariance matrix. The cell e.s.d.'s are taken into account individually in the estimation of e.s.d.'s in distances, angles and torsion angles; correlations between e.s.d.'s in cell parameters are only used when they are defined by crystal symmetry. An approximate (isotropic) treatment of cell e.s.d.'s is used for estimating e.s.d.'s involving l.s. planes.
Refinement. Refinement of *F*^2^ against ALL reflections. The weighted *R*-factor *wR* and goodness of fit *S* are based on *F*^2^, conventional *R*-factors *R* are based on *F*, with *F* set to zero for negative *F*^2^. The threshold expression of *F*^2^ > σ(*F*^2^) is used only for calculating *R*-factors(gt) *etc*. and is not relevant to the choice of reflections for refinement. *R*-factors based on *F*^2^ are statistically about twice as large as those based on *F*, and *R*- factors based on ALL data will be even larger.

### Fractional atomic coordinates and isotropic or equivalent isotropic displacement parameters (Å^2^)

**Table:**

	*x*	*y*	*z*	*U*_iso_*/*U*_eq_	
C1	0.3360 (3)	0.7691 (2)	0.1809 (2)	0.0586 (5)	
H1	0.2262	0.7682	0.1477	0.070*	
C2	0.2880 (3)	0.8977 (2)	0.2672 (3)	0.0688 (6)	
H2A	0.1797	0.9729	0.2480	0.083*	
H2B	0.2613	0.8741	0.3566	0.083*	
C3	0.4616 (3)	0.94003 (18)	0.23750 (19)	0.0500 (5)	
C4	0.5098 (3)	1.0335 (2)	0.3022 (2)	0.0609 (5)	
H4	0.4299	1.0798	0.3760	0.073*	
C5	0.6771 (3)	1.0575 (2)	0.2565 (3)	0.0705 (6)	
H5	0.7098	1.1214	0.2990	0.085*	
C6	0.7955 (3)	0.9884 (3)	0.1493 (3)	0.0755 (7)	
H6	0.9087	1.0055	0.1198	0.091*	
C7	0.7506 (3)	0.8938 (2)	0.0837 (2)	0.0715 (6)	
H7	0.8329	0.8462	0.0112	0.086*	
C8	0.5807 (3)	0.87095 (18)	0.12792 (19)	0.0529 (5)	
C9	0.4947 (3)	0.7796 (2)	0.07299 (19)	0.0657 (6)	
H9A	0.5867	0.6874	0.0489	0.079*	
H9B	0.4456	0.8222	−0.0017	0.079*	
C10	0.4294 (2)	0.51886 (17)	0.19832 (17)	0.0416 (4)	
H10A	0.5368	0.4475	0.2233	0.050*	
H10B	0.4588	0.5283	0.1063	0.050*	
C11	0.1516 (2)	0.48967 (16)	0.14342 (15)	0.0366 (4)	
C12	−0.0368 (2)	0.36456 (17)	0.28236 (17)	0.0422 (4)	
C13	0.0822 (2)	0.35258 (16)	0.37605 (15)	0.0379 (4)	
C14	0.0470 (3)	0.28777 (19)	0.49221 (17)	0.0495 (4)	
H14	−0.0524	0.2519	0.5098	0.059*	
C15	0.1577 (3)	0.27652 (19)	0.58079 (18)	0.0548 (5)	
H15	0.1350	0.2321	0.6579	0.066*	
C16	0.3030 (3)	0.33168 (19)	0.55437 (17)	0.0515 (5)	
H16	0.3775	0.3247	0.6148	0.062*	
C17	0.3401 (2)	0.39664 (18)	0.44111 (16)	0.0446 (4)	
H17	0.4386	0.4335	0.4253	0.053*	
C18	0.2296 (2)	0.40725 (16)	0.34981 (15)	0.0350 (4)	
N1	0.26417 (18)	0.47265 (13)	0.23194 (12)	0.0356 (3)	
N2	0.00901 (19)	0.43362 (14)	0.17284 (13)	0.0422 (3)	
H2	−0.0598	0.4425	0.1165	0.051*	
O1	0.40148 (17)	0.64742 (12)	0.25756 (11)	0.0487 (3)	
O2	0.17725 (17)	0.54975 (13)	0.04228 (11)	0.0490 (3)	
O3	−0.16859 (19)	0.32033 (15)	0.29619 (14)	0.0658 (4)	

### Atomic displacement parameters (Å^2^)

**Table:**

	*U*^11^	*U*^22^	*U*^33^	*U*^12^	*U*^13^	*U*^23^
C1	0.0577 (12)	0.0473 (10)	0.0821 (15)	−0.0195 (9)	−0.0355 (11)	0.0023 (10)
C2	0.0494 (11)	0.0560 (12)	0.1013 (18)	−0.0166 (10)	−0.0134 (11)	−0.0133 (11)
C3	0.0489 (10)	0.0378 (9)	0.0665 (13)	−0.0140 (8)	−0.0192 (9)	0.0032 (9)
C4	0.0620 (12)	0.0467 (11)	0.0785 (15)	−0.0201 (9)	−0.0190 (11)	−0.0048 (10)
C5	0.0794 (16)	0.0567 (12)	0.0951 (18)	−0.0362 (12)	−0.0405 (14)	0.0135 (12)
C6	0.0627 (14)	0.0710 (15)	0.102 (2)	−0.0354 (12)	−0.0199 (14)	0.0302 (14)
C7	0.0780 (15)	0.0614 (13)	0.0697 (15)	−0.0267 (12)	−0.0002 (12)	0.0189 (11)
C8	0.0673 (13)	0.0396 (9)	0.0538 (11)	−0.0183 (9)	−0.0182 (10)	0.0129 (8)
C9	0.1052 (17)	0.0496 (11)	0.0515 (12)	−0.0336 (11)	−0.0238 (12)	0.0084 (9)
C10	0.0373 (9)	0.0488 (10)	0.0457 (10)	−0.0207 (8)	−0.0123 (7)	0.0004 (8)
C11	0.0381 (9)	0.0392 (8)	0.0378 (9)	−0.0160 (7)	−0.0143 (7)	0.0012 (7)
C12	0.0386 (9)	0.0445 (9)	0.0524 (11)	−0.0217 (8)	−0.0161 (8)	0.0050 (8)
C13	0.0387 (9)	0.0364 (8)	0.0409 (9)	−0.0130 (7)	−0.0121 (7)	0.0020 (7)
C14	0.0491 (11)	0.0502 (10)	0.0512 (11)	−0.0204 (9)	−0.0100 (9)	0.0125 (8)
C15	0.0673 (13)	0.0505 (11)	0.0427 (11)	−0.0132 (10)	−0.0155 (9)	0.0126 (8)
C16	0.0619 (12)	0.0492 (10)	0.0451 (10)	−0.0112 (9)	−0.0272 (9)	0.0035 (8)
C17	0.0461 (10)	0.0461 (10)	0.0478 (10)	−0.0165 (8)	−0.0218 (8)	0.0029 (8)
C18	0.0375 (9)	0.0334 (8)	0.0364 (9)	−0.0109 (7)	−0.0139 (7)	0.0002 (6)
N1	0.0368 (7)	0.0413 (7)	0.0367 (7)	−0.0192 (6)	−0.0152 (6)	0.0041 (6)
N2	0.0429 (8)	0.0546 (8)	0.0423 (8)	−0.0257 (7)	−0.0232 (6)	0.0079 (7)
O1	0.0582 (8)	0.0530 (7)	0.0509 (7)	−0.0340 (6)	−0.0203 (6)	0.0023 (6)
O2	0.0567 (8)	0.0639 (8)	0.0401 (7)	−0.0323 (6)	−0.0222 (6)	0.0146 (6)
O3	0.0605 (8)	0.0829 (10)	0.0790 (10)	−0.0490 (8)	−0.0326 (7)	0.0265 (8)

### Geometric parameters (Å, º)

**Table:**

C1—O1	1.443 (2)	C10—N1	1.4641 (19)
C1—C9	1.525 (3)	C10—H10A	0.9700
C1—C2	1.525 (3)	C10—H10B	0.9700
C1—H1	0.9800	C11—O2	1.2247 (19)
C2—C3	1.499 (3)	C11—N2	1.3663 (19)
C2—H2A	0.9700	C11—N1	1.3725 (19)
C2—H2B	0.9700	C12—O3	1.2144 (18)
C3—C4	1.378 (3)	C12—N2	1.375 (2)
C3—C8	1.382 (3)	C12—C13	1.460 (2)
C4—C5	1.372 (3)	C13—C18	1.389 (2)
C4—H4	0.9300	C13—C14	1.393 (2)
C5—C6	1.362 (3)	C14—C15	1.370 (3)
C5—H5	0.9300	C14—H14	0.9300
C6—C7	1.377 (3)	C15—C16	1.378 (3)
C6—H6	0.9300	C15—H15	0.9300
C7—C8	1.383 (3)	C16—C17	1.371 (2)
C7—H7	0.9300	C16—H16	0.9300
C8—C9	1.495 (3)	C17—C18	1.394 (2)
C9—H9A	0.9700	C17—H17	0.9300
C9—H9B	0.9700	C18—N1	1.410 (2)
C10—O1	1.4011 (19)	N2—H2	0.8600
			
O1—C1—C9	110.81 (16)	O1—C10—H10A	109.1
O1—C1—C2	106.40 (17)	N1—C10—H10A	109.1
C9—C1—C2	105.33 (16)	O1—C10—H10B	109.1
O1—C1—H1	111.3	N1—C10—H10B	109.1
C9—C1—H1	111.3	H10A—C10—H10B	107.8
C2—C1—H1	111.3	O2—C11—N2	121.04 (15)
C3—C2—C1	104.25 (17)	O2—C11—N1	122.58 (14)
C3—C2—H2A	110.9	N2—C11—N1	116.37 (14)
C1—C2—H2A	110.9	O3—C12—N2	120.33 (16)
C3—C2—H2B	110.9	O3—C12—C13	124.94 (16)
C1—C2—H2B	110.9	N2—C12—C13	114.72 (13)
H2A—C2—H2B	108.9	C18—C13—C14	119.96 (16)
C4—C3—C8	120.32 (18)	C18—C13—C12	119.93 (15)
C4—C3—C2	129.21 (19)	C14—C13—C12	120.11 (15)
C8—C3—C2	110.47 (17)	C15—C14—C13	120.45 (17)
C5—C4—C3	119.3 (2)	C15—C14—H14	119.8
C5—C4—H4	120.4	C13—C14—H14	119.8
C3—C4—H4	120.4	C14—C15—C16	119.26 (17)
C6—C5—C4	120.5 (2)	C14—C15—H15	120.4
C6—C5—H5	119.8	C16—C15—H15	120.4
C4—C5—H5	119.8	C17—C16—C15	121.49 (18)
C5—C6—C7	121.1 (2)	C17—C16—H16	119.3
C5—C6—H6	119.4	C15—C16—H16	119.3
C7—C6—H6	119.4	C16—C17—C18	119.68 (16)
C6—C7—C8	118.7 (2)	C16—C17—H17	120.2
C6—C7—H7	120.6	C18—C17—H17	120.2
C8—C7—H7	120.6	C13—C18—C17	119.16 (15)
C3—C8—C7	120.04 (19)	C13—C18—N1	119.53 (14)
C3—C8—C9	110.30 (17)	C17—C18—N1	121.31 (14)
C7—C8—C9	129.6 (2)	C11—N1—C18	122.06 (13)
C8—C9—C1	104.28 (16)	C11—N1—C10	117.92 (13)
C8—C9—H9A	110.9	C18—N1—C10	119.96 (13)
C1—C9—H9A	110.9	C11—N2—C12	127.31 (14)
C8—C9—H9B	110.9	C11—N2—H2	116.3
C1—C9—H9B	110.9	C12—N2—H2	116.3
H9A—C9—H9B	108.9	C10—O1—C1	114.24 (13)
O1—C10—N1	112.53 (13)		
			
O1—C1—C2—C3	96.00 (19)	C14—C15—C16—C17	−0.5 (3)
C9—C1—C2—C3	−21.7 (2)	C15—C16—C17—C18	−0.2 (3)
C1—C2—C3—C4	−167.6 (2)	C14—C13—C18—C17	−0.2 (2)
C1—C2—C3—C8	12.9 (2)	C12—C13—C18—C17	179.10 (15)
C8—C3—C4—C5	0.2 (3)	C14—C13—C18—N1	180.00 (15)
C2—C3—C4—C5	−179.3 (2)	C12—C13—C18—N1	−0.7 (2)
C3—C4—C5—C6	−0.9 (3)	C16—C17—C18—C13	0.6 (2)
C4—C5—C6—C7	0.4 (3)	C16—C17—C18—N1	−179.66 (15)
C5—C6—C7—C8	0.8 (3)	O2—C11—N1—C18	177.94 (15)
C4—C3—C8—C7	1.0 (3)	N2—C11—N1—C18	−3.1 (2)
C2—C3—C8—C7	−179.43 (19)	O2—C11—N1—C10	−4.9 (2)
C4—C3—C8—C9	−178.03 (17)	N2—C11—N1—C10	174.07 (14)
C2—C3—C8—C9	1.6 (2)	C13—C18—N1—C11	2.8 (2)
C6—C7—C8—C3	−1.4 (3)	C17—C18—N1—C11	−176.99 (15)
C6—C7—C8—C9	177.3 (2)	C13—C18—N1—C10	−174.32 (14)
C3—C8—C9—C1	−15.3 (2)	C17—C18—N1—C10	5.9 (2)
C7—C8—C9—C1	165.8 (2)	O1—C10—N1—C11	103.02 (16)
O1—C1—C9—C8	−92.08 (18)	O1—C10—N1—C18	−79.75 (18)
C2—C1—C9—C8	22.6 (2)	O2—C11—N2—C12	−179.57 (16)
O3—C12—C13—C18	179.98 (17)	N1—C11—N2—C12	1.4 (2)
N2—C12—C13—C18	−0.9 (2)	O3—C12—N2—C11	179.69 (17)
O3—C12—C13—C14	−0.7 (3)	C13—C12—N2—C11	0.5 (2)
N2—C12—C13—C14	178.46 (16)	N1—C10—O1—C1	−93.88 (17)
C18—C13—C14—C15	−0.5 (3)	C9—C1—O1—C10	−73.73 (18)
C12—C13—C14—C15	−179.80 (17)	C2—C1—O1—C10	172.28 (14)
C13—C14—C15—C16	0.8 (3)		

### Hydrogen-bond geometry (Å, º)

**Table:**

*D*—H···*A*	*D*—H	H···*A*	*D*···*A*	*D*—H···*A*
N2—H2···O2^i^	0.86	2.06	2.9106 (18)	169
C9—H9*A*···O2^ii^	0.97	2.56	3.527 (3)	173
C16—H16···O1^iii^	0.93	2.47	3.378 (2)	166
C10—H10*A*···O3^iv^	0.97	2.46	3.404 (2)	165
C5—H5···O3^v^	0.93	2.47	3.314 (2)	151

Symmetry codes: (i) −*x*, −*y*+1, −*z*; (ii) −*x*+1, −*y*+1, −*z*; (iii) −*x*+1, −*y*+1, −*z*+1; (iv) *x*+1, *y*, *z*; (v) *x*+1, *y*+1, *z*.
